# Intersecting ethnic and socioeconomic inequalities in infant mortality in England, 2007–2019

**DOI:** 10.1136/archdischild-2023-326619

**Published:** 2024-07-01

**Authors:** Oluwaseun B. Esan, Paul Norman, Philip McHale, Dougal Hargreaves, Hanna Creese, G J Melendez-Torres, David Taylor-Robinson

**Affiliations:** 1 Department of Public Health Policy and Systems, University of Liverpool, Liverpool, UK; 2 NIHR School for Public Health Research, Newcastle upon Tyne, UK; 3 School of Geography, University of Leeds, Leeds, UK; 4 Department of Primary Care and Public Health, Imperial College London, London, UK; 5 Medical School, University of Exeter, Exeter, UK

**Keywords:** Epidemiology, Mortality, Paediatrics, Child Health

 There are stark socioeconomic and ethnic inequalities in infant mortality in the UK.^1^ Previously, we have demonstrated that rising child poverty led to an increase in the infant mortality rate (IMR) in England following a period of sustained decline.^2^ Recent estimates show stagnation in improvements in IMR and increasing inequalities over the last decade in UK nations.^3^ This study aims to determine if previously observed social patterning of IMR is consistent across all ethnic groups.

**Figure 1:**
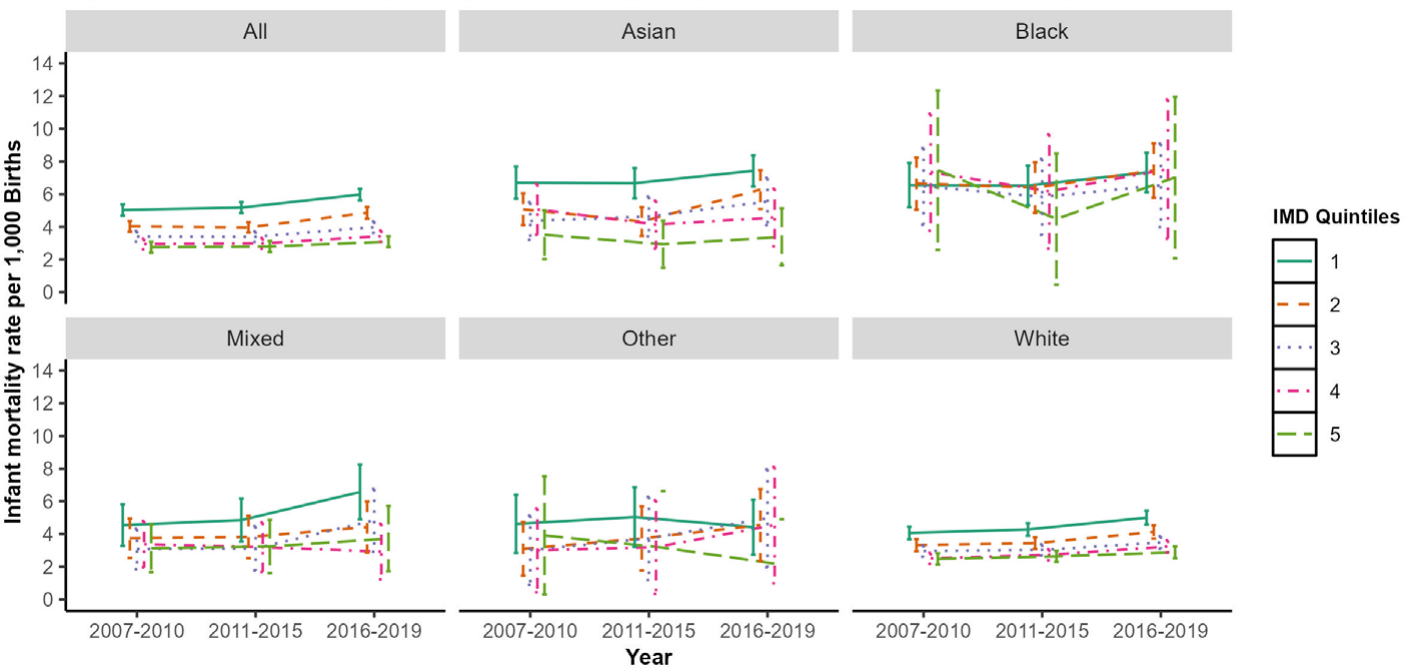
Four to 5-year infant mortality rate by Index of Multiple Deprivation (IMD) and ethnicity in England, 2007–2019. Figure 1 shows 4–5 yearly infant mortality rate by ethnic categories and IMD quintiles in England for the period 2007–2019. IMD is a relative area-level measure of deprivation based on income, employment, education, health, housing, crime and the living environment of approximately 1000–1500 households. Office for National Statistics-published IMD deciles were collapsed to IMD quintiles due to small numbers for some ethnic groups. IMD quintiles: 1—most deprived and 5—least deprived. Error bars show corresponding 95% CIs.

**Figure 2:**
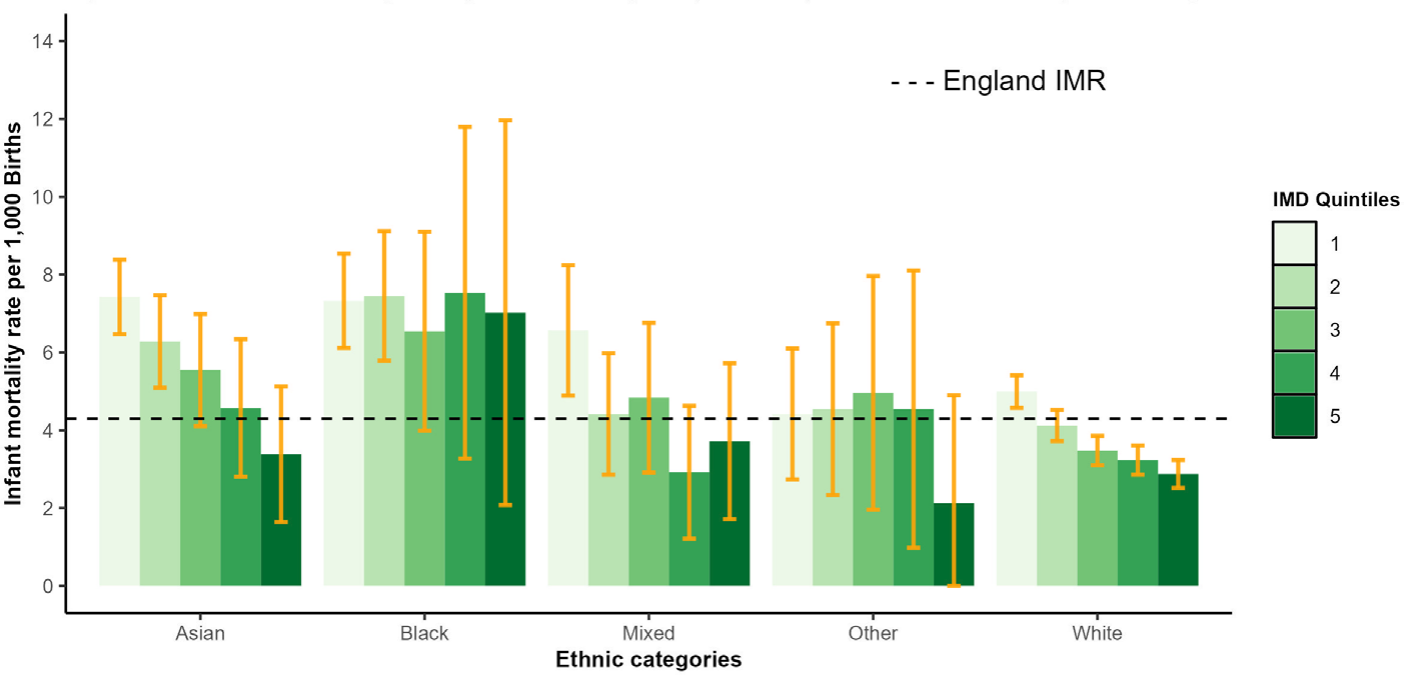
Four-year infant mortality rate (IMR) by Index of Multiple Deprivation (IMD) and ethnicity in England, 2016–2019. Figure 2 shows the IMR by ethnic categories and IMD quintiles for the most recent period (2016–2019). Year groups were based on the IMD applied to the year in question (2016–2019 uses IMD-2019). IMD deciles were collapsed to IMD quintiles due to small numbers for some ethnic groups. Error bars show corresponding 95% CIs.

In this analysis, we used publicly available data on births and deaths, ethnicity of mothers and the Index of Multiple Deprivation (IMD) from 2007 to 2019. We calculated IMR by IMD quintile (Office for National Statistics (ONS) IMD deciles collapsed to quintiles) and broad ethnic categories of black (black African, black Caribbean, any other black background), Asian (Bangladeshi, Pakistani, Indian, Chinese, any other Asian background), mixed background, white (white British, white other) and any other ethnic background.^2^ In keeping with ONS methods, we used deaths registered in a year as the numerator and births registered in a year as the denominator, pooling over year bands. We present the results graphically for year trends (figure 1) and show results for the most recent period to compare social gradients within and across ethnic groups (figure 2).

Figure 1 shows a clear deprivation gradient and rising inequalities over time across most ethnic groups except for the black ethnic category for each of the year bands, with the highest IMR observed among those living in the most deprived quintile.

There are clear socioeconomic status (SES) gradients for Asian, mixed and white ethnicity categories, while for the black ethnicity category, there is no clear SES gradient with a high IMR across all SES levels (figure 2). On average, IMR for this category was nearly double the England rate across all IMD quintiles (average of 7.1 per 1000 births), although with large CIs for the most affluent groups.

There are large and intersecting SES and ethnic inequalities in IMR in England, which vary across ethnic categories. IMR is particularly high for infants of the black ethnic category regardless of deprivation, indicating that other factors beyond SES may contribute to poor outcomes and it is consistent with reported poorer maternal outcomes for black women.^4^ However, the findings are limited by using broad ethnic categories which mask heterogeneity within groups and homogenise experiences. The current data availability limits the use of granular categories. It will lead to a loss of confidentiality due to small cell counts, imprecise estimates and large CIs. Moreover, the use of crude descriptive statistics without data on known risk factors for IMR limits inference on causal explanations. These findings highlight the necessity for granular, multilevel quantitative intersectional analyses to reveal how certain ethnic groups may experience multiplicative adverse health outcomes due to the impacts of intersecting socioeconomic and ethnic inequalities. Such comprehensive analyses can capture heterogeneity across and within ethnic groups, informing clinicians on tailoring care to address the intersectional effects of these inequalities that may not follow expected social patterns.
